# Household Environmental Risk Factors Associated with *Ehrlichia* spp. Infection in Dogs from Homes with Human Rickettsiosis Exposure in Northwestern Mexico

**DOI:** 10.3390/pathogens15070694

**Published:** 2026-06-30

**Authors:** José Mario Atondo-Pacheco, Rosalino Flores-Rocha, María de J. López-López, Idalia Enríquez-Verdugo, Daniel Eduardo Zatarain, Jesús Daniel Solis-Carrasco, Nohelia Castro-del Campo, Sandra Berenice Medina-Rodríguez, Soila Maribel Gaxiola-Camacho, Nohemí Castro-del Campo

**Affiliations:** 1 Facultad de Medicina Veterinaria y Zootecnia, Universidad Autónoma de Sinaloa, Culiacán 80246, Sinaloa, Mexico; 2 Facultad de Medicina, Universidad Autónoma de Sinaloa, Culiacán 80019, Sinaloa, Mexico; 3 Laboratorio Nacional para la Investigación en Inocuidad Alimentaria, Centro de Investigación en Alimentación y Desarrollo, Culiacán 80110, Sinaloa, Mexico

**Keywords:** *Ehrlichia* spp., *Rhipicephalus sanguineus*, tick-borne diseases, household risk factors, One Health

## Abstract

Zoonotic diseases represent an increasing public health concern worldwide, particularly in endemic regions of northwestern Mexico. This study aimed to evaluate household-level environmental and behavioral risk factors associated with *Ehrlichia* spp. infection in dogs living in areas with documented human rickettsiosis cases in Culiacán, Sinaloa, Mexico. A cross-sectional study was conducted, including 105 canine blood samples collected from urban and rural areas, with previous human rickettsiosis cases reported between 2021 and 2023. Microscopic examination and PCR amplification targeting the 16S rRNA gene of *Ehrlichia* spp. were performed. Morphological detection revealed a prevalence of 33.33%, while molecular analysis showed a prevalence of 43.83%. Risk factors significantly associated with infection included household waste, soil-type environments, free-roaming behavior identified through epidemiological analysis, and limited knowledge of tick-borne diseases among dog owners. These findings provide evidence of *Ehrlichia* spp. infection in dogs from areas with documented human rickettsiosis cases and highlight the value of dogs as indicators of household-level exposure to tick-borne pathogens in endemic communities.

## 1. Introduction

Ehrlichiosis is a tick-borne infectious disease caused by an obligate intracellular Gram-negative bacterium of the order Rickettsiales, family Anaplasmataceae. In dogs, two main clinical forms have been described: granulocytic ehrlichiosis caused by *Ehrlichia ewingii* and monocytic ehrlichiosis, primarily caused by *Ehrlichia canis* and, less frequently, *Ehrlichia chaffeensis* [1,2]. Canine monocytic ehrlichiosis is considered the most clinically relevant form and may present acute, subclinical, or chronic stages depending on host immune response and infection dynamics; clinical manifestations commonly include fever, lethargy, thrombocytopenia, anemia, and hemorrhagic disorders, representing an important veterinary health concern in endemic regions [1].

The geographical expansion of tick populations and the pathogens they carry has been associated with environmental changes, urbanization, climate variability, and the close coexistence of humans and domestic animals [3]. The brown dog tick, *Rhipicephalus sanguineus*, is widely distributed in tropical and subtropical regions and is highly adapted to peridomestic environments, including human dwellings [4,5]. Its capacity to complete its life cycle indoors increases the risk of exposure for both dogs and humans, particularly in warm climatic regions such as northwestern Mexico. These ecological conditions favor the maintenance and transmission of tick-borne pathogens at the human–animal interface.

In Mexico, tick-borne zoonotic diseases, such as rickettsiosis, represent an emerging public health problem, especially in northwestern states, including Sinaloa, where environmental conditions facilitate vector proliferation and transmission cycles [6,7]. Previous studies have documented the presence of *Rhipicephalus sanguineus* in domestic dogs from urban and rural environments in Sinaloa and have highlighted its role as a vector of pathogens of veterinary and medical importance [4,5,8]. However, the epidemiological relationship between canine exposure to *Ehrlichia* spp. and households with confirmed human rickettsiosis cases remains insufficiently explored in this region.

In northwestern Mexico, particularly in the state of Sinaloa, tick-borne diseases represent an important veterinary and public health concern. Previous studies have documented the widespread presence of *Rhipicephalus sanguineus* in domestic dogs from urban and rural communities and have documented the presence of *Ehrlichia canis* in canine populations from the region [4,5,8,9,10]. In addition, human rickettsiosis caused by *Rickettsia rickettsii* has been repeatedly reported in northwestern Mexico, where environmental and socioeconomic conditions favor the persistence of tick vectors and increase the risk of pathogen transmission [7,11,12]. These findings highlight the epidemiological importance of domestic environments as potential sites of interaction among vectors, animal hosts, and humans.

Previous studies conducted in Sinaloa have mainly focused on estimating the prevalence of *Ehrlichia* spp. infection in dogs, describing clinical manifestations, identifying tick vectors, or detecting pathogen circulation in canine and tick populations [8,10]. However, limited information is available regarding household-level environmental and behavioral factors associated with *Ehrlichia* spp. infection in dogs living in areas with documented human exposure to tick-borne diseases. Furthermore, the role of domestic environmental conditions, sanitation practices, and owner-related factors in shaping exposure risk remains insufficiently characterized in endemic communities of northwestern Mexico.

Dogs residing in households with a history of human rickettsiosis cases may be exposed to environmental conditions that favor tick infestation and pathogen transmission. Factors such as free-roaming behavior, household sanitation, soil characteristics, and owner awareness of tick-borne diseases may influence the risk of *Ehrlichia* spp. infection in dogs and contribute to the maintenance of tick populations within domestic environments; understanding these factors may help identify household-level environmental and behavioral factors associated with increased exposure to tick-borne pathogens in endemic areas [13,14].

Therefore, this study aimed to estimate the prevalence of *Ehrlichia* spp. infection in domestic dogs living in households with previous human rickettsiosis cases or located in neighboring households within the same affected areas in Culiacán, Sinaloa, Mexico, and to identify household-level environmental and behavioral factors associated with infection. By exploring these factors, the study seeks to contribute to a better understanding of environmental conditions associated with exposure to tick-borne pathogens in endemic communities from a One Health perspective.

## 2. Materials and Methods

### 2.1. Study Area and Sampling Strategy

Blood samples were collected from owned domestic dogs living in urban and rural areas of Culiacán, Sinaloa, Mexico (Figure 1). Sampling locations were selected using records of confirmed human rickettsiosis cases reported by the Secretaría de Salud of the state of Sinaloa between 2021 and 2023. These records were used as geographic reference points for identifying areas with documented human exposure to tick-borne diseases.

Dogs were sampled from households with previously reported human rickettsiosis cases when available, as well as from neighboring households located within the same streets or surrounding areas. Therefore, the study focused on dogs living in areas with documented human rickettsiosis cases rather than exclusively on households with confirmed human cases.

Field sampling and questionnaire administration were conducted between June 2023 and May 2024.

Sampling was conducted in both urban and rural communities of Culiacán, including neighborhoods and localities located within areas previously identified as having reported human rickettsiosis cases. Only owned dogs whose owners voluntarily agreed to participate and provided informed consent were included in the study.

When more than one dog was present in a household, all available dogs were eligible for sampling regardless of the presence of ticks, sanitary conditions, or clinical status. A total of 105 dogs were included using a convenience sampling approach.

### 2.2. Sample Collection

Blood samples (3–5 mL) were obtained from each dog by cephalic or jugular venipuncture using vacuum tubes containing EDTA as an anticoagulant. Samples were transported at 4 °C to the Parasitology Laboratory of the Facultad de Medicina Veterinaria y Zootecnia, Universidad Autónoma de Sinaloa, where they were processed within 24 h for microscopic and molecular analyses. Dog owners provided informed consent prior to sample collection and participation in the epidemiological survey. Written informed consent was obtained using a standardized consent form signed by the owner before sample collection.

### 2.3. Microscopic Identification

Peripheral blood smears were prepared from each blood sample and stained using a Hemocolor^®^ staining kit (Hycel^®^, Mexico City, Mexico) according to the manufacturer’s instructions. After staining and drying, slides were examined under light microscopy using a 100× oil immersion objective to identify intracytoplasmic morulae compatible with *Ehrlichia* spp. infection within leukocytes, following previously described morphological criteria for canine ehrlichiosis [15]. A sample was considered positive when at least one morula compatible with *Ehrlichia* spp. was observed. Following microscopic examination, blood samples were stored at −20 °C until DNA extraction.

### 2.4. DNA Extraction and Quantification

Genomic DNA was extracted from the 105 blood samples using the DNeasy^®^ Blood & Tissue Kit (QIAGEN, Hilden, Germany) following the manufacturer’s instructions.

DNA was quantified using a NanoDrop One spectrophotometer (Thermo Fisher Scientific, Waltham, MA, USA). Only samples with DNA concentrations ≥ 16 ng/µL were included for PCR analysis to ensure adequate amplification.

### 2.5. Molecular Detection by PCR

Molecular detection of *Ehrlichia* spp. was performed by conventional PCR targeting a conserved region of the 16S rRNA gene using primers ECC (5′-AGAACGAACGCTGGCGGCAAGCC-3′) and ECB (5′-CGTATTACCGCGGCTGCTGGC-3′), which have been previously used and validated for the molecular detection of *Ehrlichia* spp. in canine blood samples [16,17].

PCR reactions were performed in a final volume of 25 µL containing 12.5 µL of PCR Master Mix (Bioline^®^, London, UK), 2 µL of each primer, and 8.5 µL of template DNA. Amplification was carried out using a T100™ Thermal Cycler (Bio-Rad Laboratories^®^, Hercules, CA, USA) under the following conditions: initial denaturation at 94 °C for 1 min, followed by 30 amplification cycles consisting of denaturation at 94 °C for 1 min, annealing at 55 °C for 2 min, and extension at 72 °C for 2 min, with a final extension at 72 °C for 5 min. The ECC and ECB primers have been extensively validated and widely used for genus-level detection of *Ehrlichia* spp. in canine blood samples.

PCR products were separated by electrophoresis on 1.5% agarose gels stained with GelRed® nucleic acid stain (Biotium, Fremont, CA, USA) and visualized using a Labnet UV Transilluminator ENDURO (Labnet International, Inc., Edison, NJ, USA). Samples producing an amplicon of approximately 480 bp were considered positive for *Ehrlichia* spp. A positive control and a no-template negative control were included in each PCR run. The negative control contained nuclease-free water instead of template DNA and showed no amplification, indicating the absence of detectable PCR contamination during the amplification assays.

### 2.6. Risk Factor Assessment

An epidemiological questionnaire was administered to dog owners to evaluate environmental and behavioral variables potentially associated with *Ehrlichia* spp. infection (Figure 2). Information was collected regarding household characteristics, owner-related factors, and canine management practices.

Thirteen variables were included in the analysis: sex of the dog (male/female), presence of ticks (yes/no), sleeping location (inside the house, patio, doghouse, or street), type of soil where the dog lived (concrete floor, soil, grass, or mixed surfaces), free access to the street (yes/no), presence of waste around the household exterior (yes/no), health status of the dog (poor, fair, good, or excellent), presence of individuals under 18 years old in the household (yes/no), socioeconomic level of the household, educational level of the owner (no formal education, primary school, secondary school, high school, or university), knowledge about tick-borne diseases (yes/no), occupation of the owner, and whether the dog was regularly walked outside the household (yes/no).

The presence of ticks was determined by direct visual inspection of each dog at the time of sampling and was recorded as present or absent in the epidemiological questionnaire.

Questionnaires were completed during household visits at the time of sample collection and were used to identify factors potentially associated with *Ehrlichia* spp. infection.

### 2.7. Statistical Analysis

Associations between *Ehrlichia* spp. infection and potential risk factors were evaluated using the chi-square test. Variables with *p*-values lower than 0.05 were considered statistically significant.

Univariate logistic regression was performed to estimate odds ratios (ORs) and 95% confidence intervals (CIs) for significant variables using RStudio version 2024.04.2 (Posit Software, Boston, MA, USA). and the EpiR statistical package.

Prevalence estimates of *Ehrlichia* spp. were calculated using the following epidemiological formula [11]:

Prevalence = (a/(a + b)) × k

where

a = number of positive animals during the study period;

a + b = total number of animals examined during the same period;

k = constant multiplier equal to 100.

## 3. Results

### 3.1. Morphological Identification in Blood

A total of 105 canine blood samples were analyzed from households located in or near residences with confirmed human rickettsiosis cases in Culiacán, Sinaloa, Mexico. Morphological examination identified 35 samples positive for *Ehrlichia* spp. infection (Figure 3), corresponding to a prevalence of 33.33%. The distribution of positive samples by locality is presented in Table 1.

To evaluate potential associations between morphological detection of *Ehrlichia* spp. and environmental and behavioral variables, 13 potential risk factors were analyzed using the chi-square test in RStudio (2024). Of these, two variables showed statistically significant associations: the dog’s sleeping location and the dog’s free access to the street (Table 2).

Variables showing statistically significant associations in the chi-square analysis were subsequently evaluated using univariate logistic regression to estimate odds ratios (Table 3). Dogs with free access to the street showed significantly higher odds of *Ehrlichia* spp. infection (OR = 4.89; 95% CI: 1.06–22.63; *p* = 0.0300). In contrast, sleeping location was not significantly associated with infection in the logistic regression model (OR = 1.22; 95% CI: 0.50–2.97; *p* = 0.8240).

### 3.2. Molecular Identification by PCR

Of the 105 collected samples, 73 met the DNA concentration criterion (≥16 ng/µL) established for inclusion in PCR analysis. Of these, 32 samples showed amplification of the expected 480 bp fragment (Figure 4) and were considered positive for *Ehrlichia* spp., corresponding to a prevalence of 43.83%.

Chi-square analysis was performed to evaluate associations between PCR positivity and the variables of interest, considering *p* < 0.05 as statistically significant. Significant associations were observed for the type of soil where the dog lived (*p* = 0.0334), the presence of waste around the household exterior (*p* = 0.0058) (Figure 5), and the owners’ knowledge about tick-borne diseases (*p* = 0.0468) (Table 4).

To continue the analysis, risk factors were evaluated using univariate logistic regression. Univariate logistic regression analysis showed that dogs living in households with earthen floors had higher odds of *Ehrlichia* spp. infection than those living in households with concrete floors (OR = 3.11; 95% CI: 1.18–8.19; *p* = 0.0310). Likewise, the presence of waste around the household exterior was associated with increased odds of infection (OR = 4.67; 95% CI: 1.65–13.21; *p* = 0.0030) (Table 5).

Owners who reported no knowledge of tick-borne diseases showed higher odds of *Ehrlichia* spp. infection in their dogs compared with those who reported previous knowledge of these diseases (OR = 3.77; 95% CI: 1.15–12.35; *p* = 0.0440) (Table 6).

## 4. Discussion

In the present study, a prevalence of 33.33% was observed through morphological identification of *Ehrlichia* spp. These findings are consistent with previous studies conducted in Sinaloa, Mexico. Sosa-Gutiérrez et al. [10] reported a prevalence of 40.1% based on blood smear examination in dogs with clinical suspicion of canine monocytic ehrlichiosis, supporting the frequent occurrence of *Ehrlichia*-compatible morulae in dogs from the region. Molecular evidence of *Ehrlichia canis* circulation in Sinaloa has also been documented by Sosa-Gutiérrez et al. [8], who detected the pathogen by PCR in naturally infected dogs. More recently, Gurrola-Ramírez et al. [18] detected *Ehrlichia canis* in ectoparasites collected from domestic dogs in Sinaloa, further supporting the occurrence of *Ehrlichia* spp. infection in the region. Similar prevalence values have also been reported in other endemic areas of Latin America, including Colombia [19] and Venezuela [20], where environmental conditions favor the persistence of tick populations and facilitate pathogen transmission.

Dogs with free access to the street showed an odds ratio of 4.89 for *Ehrlichia* spp. infection. These findings are consistent with those reported by Zazueta et al. [12], who identified domestic dogs living in peridomestic environments with unrestricted outdoor access as an important source of infected ticks, representing a risk for both dogs and their owners. Similarly, Hernández-Ramírez et al. [21] reported that 53% of sampled dogs in Sinaloa had contact with street environments, highlighting free-roaming dogs as potential reservoirs of zoonotic pathogens. Medina et al. [22] also reported that interaction with other dogs outside the household represented a significant risk factor, with an odds ratio of 2.872.

Martínez et al. [23] analyzed *Rhipicephalus sanguineus* ticks collected from dogs in rural communities and reported the presence of *Rickettsia* spp. in 25% (3/12) of the analyzed ticks using PCR. They also suggested that the accumulation of organic and inorganic waste and poor hygiene conditions around households favor the presence of tick vectors. These findings are consistent with the present study, which identified soil-type environments and the presence of waste around households as significant environmental risk factors associated with *Ehrlichia* spp. infection in dogs. These environmental conditions may provide favorable microhabitats for tick survival by increasing humidity, providing shelter, and facilitating contact between dogs, ticks, and other animals that may contribute to pathogen circulation.

Another relevant finding of the present study was an odds ratio of 3.11 associated with soil-type environments where dogs lived. Similar results were reported by Medina et al. [22], who identified household environments in soil-type areas as a significant risk factor in marginalized neighborhoods of Culiacán, Sinaloa, with an odds ratio of 4.103.

Molecular detection identified *Ehrlichia* spp. DNA in 43.83% of the analyzed samples, a prevalence higher than that obtained by blood smear examination. This difference was expected because PCR is generally considered more sensitive than microscopic examination, particularly in animals with low circulating bacterial loads or subclinical infections. Similar studies have demonstrated the usefulness of molecular methods for detecting *Ehrlichia canis* in naturally infected dogs and ticks, allowing the identification of infected animals in which morulae may not be readily observed in peripheral blood smears [16,17]. The relatively high molecular prevalence observed in the present study further supports the presence of *Ehrlichia* spp. infection in canine populations from areas with documented exposure to tick-borne pathogens.

The identification of environmental and behavioral risk factors at the household level highlights the importance of domestic ecological conditions associated with *Ehrlichia* spp. infection in endemic communities. Because the sampled dogs originated from households located in or near residences with documented human rickettsiosis cases, the detection of *Ehrlichia* spp. in dogs indicates the occurrence of tick-borne pathogen exposure within these environments. These findings highlight the relevance of household-level prevention measures and may contribute to future surveillance strategies in areas with documented human exposure to tick-borne diseases.

An additional relevant finding of the present study was an odds ratio of 3.77 for the lack of knowledge about tick-borne diseases, affecting both pets and their owners. Previous studies have shown that increased awareness of tick-borne diseases contributes to improved preventive practices. Septfons et al. [24] demonstrated that greater knowledge of tick-borne diseases increases preventive behavior among exposed populations. Similarly, Sidhik et al. [14] reported that 65.7% of individuals in a marginalized community in India were regularly exposed to tick bites and that 52.6% did not take preventive measures due to a lack of knowledge about tick-borne diseases. Beck et al. [25] found that individuals who perceived tick-borne diseases as common in their communities were more likely to adopt preventive behaviors than those who did not perceive them as a health concern.

This study has some limitations that should be considered when interpreting the results. First, molecular detection was performed using a genus-level PCR assay with ECC and ECB primers, which have been extensively validated for the detection of *Ehrlichia* spp. in canine blood samples. However, sequencing of amplification products was not performed; therefore, species-level identification could not be confirmed, and the molecular findings should be interpreted as evidence of *Ehrlichia* spp. DNA detection at the genus level. Future studies, including sequencing of representative positive amplicons, are warranted to characterize circulating *Ehrlichia* species and confirm the identity of the amplified products. Second, only samples with DNA concentrations ≥ 16 ng/µL were included in the molecular analysis to ensure adequate amplification, reducing the number of samples available for PCR testing. Finally, the use of convenience sampling may limit the extrapolation of these findings to the broader canine population of Culiacán. Despite these limitations, the study provides valuable information regarding environmental and behavioral factors associated with *Ehrlichia* spp. infection in areas with documented human exposure to tick-borne diseases.

## 5. Conclusions

*Ehrlichia* spp. infection was detected in dogs from households located in or near residences with documented human rickettsiosis cases in Culiacán, Sinaloa, Mexico. Morphological examination identified a prevalence of 33.33%, whereas molecular detection identified a prevalence of 43.83%, supporting the greater sensitivity of PCR for detecting infected animals. Environmental and behavioral factors, including free access of dogs to the street, soil-type environments, the presence of waste around households, and limited owner knowledge regarding tick-borne diseases, were associated with increased odds of *Ehrlichia* spp. infection. These findings highlight the importance of household environmental conditions in the transmission dynamics of tick-borne pathogens in endemic communities. The results support the value of dogs as indicators of household-level exposure to tick-borne pathogens and may contribute to future surveillance and prevention strategies in areas with documented human exposure to tick-borne diseases.

## Figures and Tables

**Figure 1:**
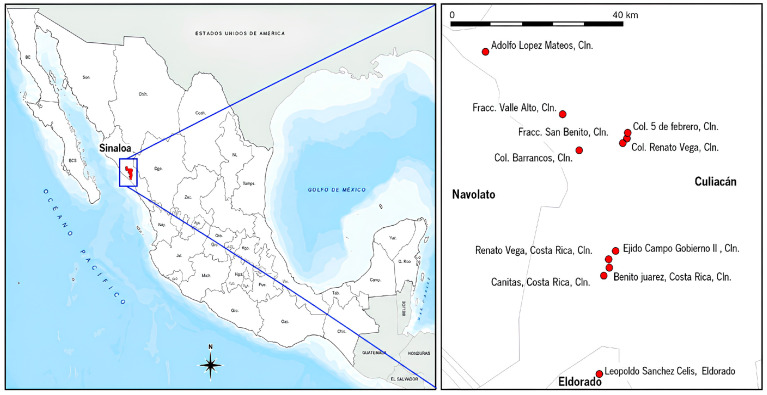
Geographic location of the study area in Sinaloa, Mexico. The left panel shows the location of Sinaloa within Mexico, and the right panel shows the sampling localities in the municipalities of Culiacán, Navolato, and Eldorado. Red points indicate study localities. Map created by the authors using QGIS and publicly available cartographic data from INEGI (Mexico).

**Figure 2:**
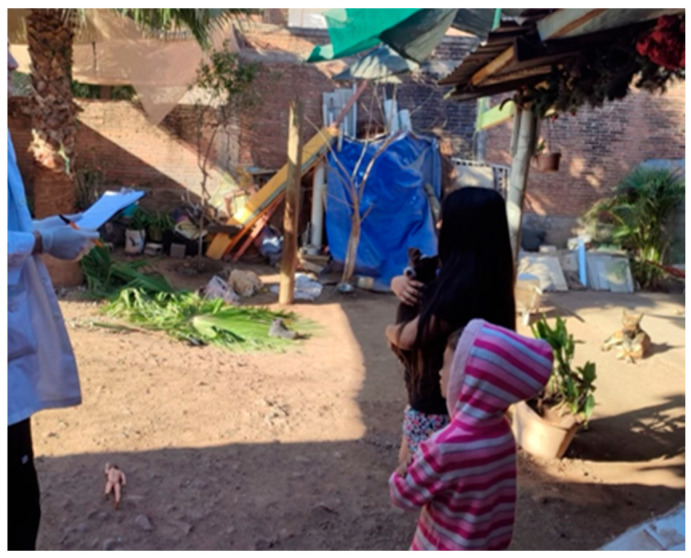
Application of epidemiological surveys to dog owners during household visits for the assessment of risk factors associated with *Ehrlichia* spp. infection.

**Figure 3:**
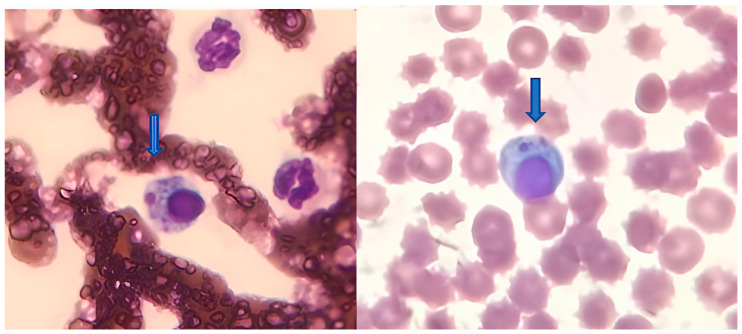
Intracytoplasmic morulae compatible with *Ehrlichia* spp. observed in monocytes from peripheral blood smears of sampled dogs (arrows). Hemocolor^®^ stain. Images were obtained using light microscopy with a 100× oil immersion objective.

**Figure 4:**
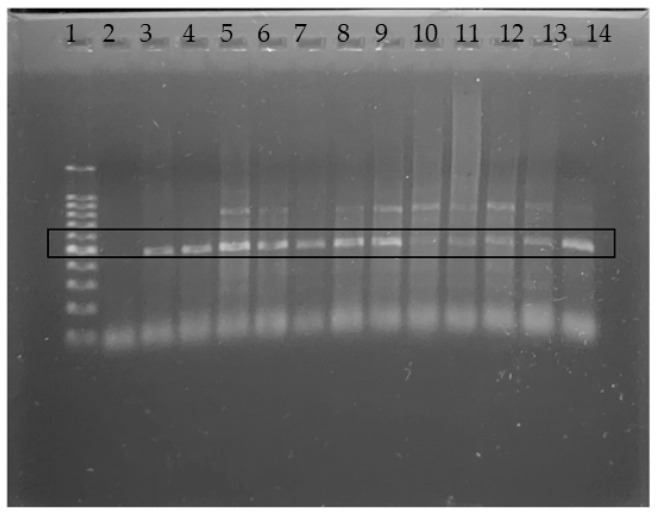
Agarose gel electrophoresis (1.5%) showing amplification of a 480 bp fragment of the 16S rRNA gene of *Ehrlichia* spp. by conventional PCR. Lane 1: 100 bp DNA ladder; Lane 2: no-template negative control; Lanes 3–13: positive canine samples; Lane 14: positive control.

**Figure 5:**
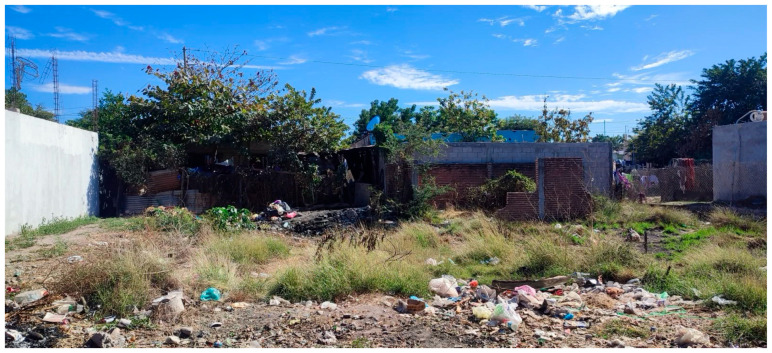
Representative household environment showing waste accumulation around the exterior of the residence, a factor significantly associated with *Ehrlichia* spp. infection in dogs according to PCR-based analysis.

**Table 1:** Results of microscopic examination for the detection of *Ehrlichia* spp. in canine blood samples.

Neighborhood	Locality	Total Samples	*Ehrlichia* spp.
San Benito	Culiacán	8	1
5 de Febrero	Culiacán	13	4
Renato Vega	Culiacán	10	6
Benito Juárez Norte, Cañitas y Constituyentes	Costa Rica, Culiacán	7	3
Renato Vega	Costa Rica, Culiacán	13	6
Barrancos	Culiacán	10	4
Valle Alto	Culiacán	6	1
Tamarindo	Culiacán	12	3
Campo Gobierno II	Culiacán	13	1
Adolfo Sánchez Celis	Eldorado	13	6
Total		105	35

**Table 2:** Analysis of blood smear results and variables of interest using the chi-square test (*p* < 0.05).

Risk Factor	*p*-Value
Sex of the dog	0.1078
Presence of ticks	0.0827
Sleeping location of the dog	0.0032
Type of soil where the dog lives	0.4450
Free access for the dog to the street	0.0300
Presence of waste around the household exterior	1.0000
Health status of the dog	0.1084
Presence of individuals under 18 years old in the household	1.0000
Socioeconomic level	0.3766
Educational level of the owner	0.4189
Knowledge about tick-borne diseases	0.5850
Occupation of the owner	0.2325
Dog walking activity	0.1960

**Table 3:** Odds ratios obtained from blood smear results for risk factors associated with sleeping location and free access to the street.

Risk Factor	Odds Ratio	95% CI	*p*-Value
Sleeping location			
House	Reference		
Street	1.22	0.50–2.97	0.8240
Free access to the street			
No	Reference		
Yes	4.89	1.06–22.63	0.0300

**Table 4:** Analysis of PCR results and variables of interest using the chi-square test, showing associations with soil type and presence of waste outside the household.

Risk Factor	*p*-Value
Sex of the dog	0.2802
Presence of ticks	0.5456
Sleeping location of the dog	0.9812
Type of soil where the dog lives	0.0334
Free access for the dog to the street	0.1540
Presence of waste around the household exterior	0.0058
Health status of the dog	0.644
Presence of individuals under 18 years old in the household	1.0000
Socioeconomic level	0.1647
Educational level of the owner	0.0841
Knowledge about tick-borne diseases	0.0468
Occupation of the owner	0.2802
Dog walking activity	0.6440

**Table 5:** Odds ratios obtained from PCR results for risk factors associated with soil type and household hygiene conditions in canine samples from Culiacán, Sinaloa, Mexico.

Risk Factor	Odds Ratio	95% CI	*p*-Value
Soil type			
Concrete floor	Reference		
Earthen floor	3.11	1.18–8.19	0.0310
Presence of waste around the household exterior			
No	Reference		
Yes	4.67	1.65–13.21	0.0030

**Table 6:** Odds ratios obtained from PCR results for risk factors associated with knowledge about tick-borne diseases.

Risk Factor	Odds Ratio	95% CI	*p*-Value
Knowledge about tick-borne diseases			
Yes	Reference		
No	3.77	1.15–12.35	0.0440

## Data Availability

The original contributions presented in this study are included in the article. Further inquiries can be directed to the corresponding authors.
